# Evaluation of LiDAR devices and data to conduct terrestrial surveys in favelas

**DOI:** 10.1038/s41597-026-07222-2

**Published:** 2026-05-22

**Authors:** Stefania Dimitrov, Fabio Duarte, Cezar Lima Ferreira Barbalho, Silvia Maria Ferreira, Carlo Ratti, Angélica Aparecida Tanus Benatti Alvim

**Affiliations:** 1 Senseable City Lab, Department of Urban Studies and Planning, Massachusetts Institute of Technology, Cambridge, USA; 2 Programa de Pós-graduação em Arquitetura e Urbanismo da Universidade Presbiteriana Mackenzie, São Paulo, Brazil; 3 Department of Innovation, Sondotécnica Engenharia, Rio de Janeiro, Brazil; 4 ABC Department, Politecnico di Milano, Milan, Italy

**Keywords:** Environmental impact, Civil engineering

## Abstract

Favelas (slums) are densely populated informal settlements that house over 1.1 billion people globally and represent challenging environments for spatial data acquisition and urban mapping. Their dense morphology, irregular street networks, and overlapping building structures make conventional mapping approaches difficult. These areas are often characterized by limited access to essential services such as water supply, sanitation, and waste collection, as well as insecure land tenure. Despite their scale, favelas are frequently absent from official maps, limiting their inclusion in urban planning processes. This Data Descriptor presents a terrestrial LiDAR dataset collected with five distinct devices along a standardized 360-meter survey route in Jardim Colombo, a favela in São Paulo, Brazil, covering approximately 5,685 m². The area was also surveyed using aerial photogrammetry and a total station to establish a spatial reference supporting comparison among the terrestrial LiDAR devices. A multicriteria framework evaluates device performance considering point density, completeness, spatial uniformity, and logistical constraints. These data support research in urban environments where accurate spatial representation remains a critical challenge.

## Background & Summary

The UN characterizes a favela as a settlement where less than 50% of households have access to drinking water and sanitation, combined with poor housing quality in terms of structure^1^. The Brazilian Institute of Geography and Statistics (IBGE) defines favelas as densely and irregularly occupied areas with at least 51 housing units built on land owned by others—either public or private—and typically lacking basic urban infrastructure^2^. These areas are characterized by high population density, unsanitary conditions, housing located in risk zones, irregular lots, narrow alleys, and often steep stairways.

Approximately one-quarter of the world’s urban population lives in favelas^1^, a figure that may significantly underestimate the actual population due to the lack of consistent global data^3^. In Brazil, around 16.4 million people live in favelas, which occupy an area of approximately 1,843 square km^2^ ^4^.

Although favelas occupy vast urban areas and house a significant number of dwellings, they are frequently neglected in official maps, which represent them as “unmappable” areas or blank spots^5^. This exclusion perpetuates socio-spatial invisibility, further marginalizing these communities in public policies and urban planning initiatives.

LiDAR (Light Detection and Ranging) technology has been increasingly used for urban mapping, providing precise results in capturing three-dimensional data through a laser scanning of space. However, while numerous studies have applied LiDAR in urban contexts, few have explored its application in favelas, where irregular morphology and logistical constraints pose unique challenges.

Research conducted in Brazilian favelas^6^ has demonstrated the potential of terrestrial LiDAR devices to complement aerial surveys, which are more commonly used for mapping extensive urban areas^7^. In dense informal settlements, aerial mapping techniques often fail to capture critical ground-level spatial structures because overlapping roofs and narrow circulation spaces obscure features from above. Terrestrial LiDAR surveys therefore play a complementary role by documenting street-level geometry, including circulation paths, infrastructure elements, and building entrance elevations that are not reliably visible in aerial datasets. This complementary contribution is particularly important for urban analysis and infrastructure planning in favelas, where access conditions and built morphology are highly irregular.

A systematic comparison of LiDAR devices under the challenging conditions of favelas has not yet been explored, leaving a gap in understanding how to optimize terrestrial data acquisition for planning and infrastructure provision in these environments.

This Data Descriptor presents a terrestrial LiDAR dataset collected in Jardim Colombo—a section of Paraisópolis favela complex, São Paulo, one of the largest favelas in Brazil. The dataset includes data from five distinct terrestrial LiDAR devices, acquired along a predefined survey route.

Each point cloud was evaluated based on technical quality criteria (density, completeness, and uniformity), as well as a study-specific logistical performance metric designed to capture field constraints. To ensure accurate georeferencing, the area was also surveyed by drone and a total station. Rather than ranking specific devices, this study identifies how different technical aspects influence the suitability of LiDAR devices for complex and irregular urban spaces.

These insights offer practical guidance for selecting terrestrial LiDAR technologies, supporting researchers, surveyors, and urban planners in creating accurate cartographic mappings of favelas.

Urban studies in informal settlements require spatial datasets capable of capturing fine-scale morphological features such as narrow alleys, irregular building footprints, and complex vertical structures. In dense environments such as Brazilian favelas, aerial mapping techniques often fail to capture critical ground-level spatial information due to occlusions caused by overlapping roofs and confined urban layouts. While drone-based photogrammetry provides accurate representations of rooftops, terrestrial surveys are necessary to document streets, alleys, facades, and infrastructure elements. In this context, LiDAR datasets can support applications such as urban morphology analysis, accessibility studies, and infrastructure planning. For these applications, users typically expect datasets with sufficient point density to represent small-scale structures, high completeness in occluded areas, and spatial uniformity across the surveyed environment. The evaluation framework adopted in this study was designed to assess these aspects to support the selection of appropriate LiDAR technologies for mapping complex informal settlements.

The dataset supports reuse in a range of scientific and technical applications, including urban morphology analysis, mapping methodology comparison, classification workflows, and 3D reconstruction studies adapted to constrained environments. However, the present study does not assume that point cloud quality directly determines the quality of a derived building surface model. Instead, the dataset is intended to support multiple downstream uses, depending on the specific objectives, processing strategies, and modeling assumptions adopted by future users.

## Methods

This section details the procedures used for data acquisition, processing, and quality assessment of the terrestrial LiDAR datasets collected in a favela environment. The methodology was designed to support cross-device comparisons and provide a robust basis for further research involving 3D mapping in complex urban areas.

### LiDAR device selection criteria

A predefined route within the study area was surveyed using five different terrestrial LiDAR devices (Table 1). To preserve neutrality and enable comparison, each device is referred to by a code (T01, S01, T02, T03, S02), and brand names are limited to Table 1 only.

**Table 1:** Technical specifications of the LiDAR devices evaluated in this study.

LiDAR device code used in this study	T01	S01	T02	T03	S02
**Commercial name**	**FARO Focus Premium**	**Leica BLK2GO**	**Leica RTC360**	**Matterport Pro3**	**NavVis VLX2**
**Image**					
**Model year**	2022	2019	2018	2022	2024
**Scanning platform**	Tripod terrestrial scanner	Handheld mobile scanner	Tripod terrestrial scanner	Tripod terrestrial scanner	Wearable mobile scanner
**Manufacturer-reported maximum range (m)**	200	25	130	100	50
**Wavelength (nm)**	1,553	830	1,550	904	903
**Maximum measurement rate (points/s)**	2,000,000	420,000	2,000,000	100,000	1,200,000
**Manufacturer-reported accuracy**	±2 mm at 10 m; ±3.5 mm at 25 m	6–15 mm (relative)	±1.9 mm at 10 m	±20 mm at 10 m	±6 mm (local)
**Field of view (vertical × horizontal, °)**	300 ×360	270 ×360	300 ×360	295 ×360	360 ×360
**Camera configuration**	1 integrated RGB camera (panoramic capture)	3 RGB cameras used for image capture and SLAM	3 RGB cameras used for panoramic HDR imaging	1 RGB camera with rotational panoramic capture	4 RGB cameras (multi-camera panoramic system)
**Camera resolution (MP)**	266 (panoramic output)	12	36	134.2	4 ×20
**Weight (kg)**	4.4	0.775	5.35	4.6	8.68
**IP rating**	IP54	IP54	IP54	IP43	IP42
**Operating temperature (°C)**	+5 to +40	0 to +40	−5 to +40	0 to +40	0 to +40

Source: Technical specifications reported by the device manufacturers^11–15^.

Note: Manufacturer-reported accuracy values follow different measurement definitions depending on the device and are provided for descriptive purposes only. Values may represent absolute ranging accuracy, relative accuracy, or local accuracy as defined by each manufacturer. Measurement rate values may refer either to point acquisition rate or equivalent scanning rate depending on the device architecture. Maximum range values correspond to manufacturer specifications and may vary according to sensor architecture.

All devices were either loaned or rented for testing purposes, and no commercial partnerships or restrictions influenced their use.

To ensure diversity in data acquisition conditions and enable comparative evaluation, the device selection followed four specific criteria:

1. Number of Channels: Single-channel, and multi-channel
2. Range: Short-range (<60 m), Medium-range (60–200 m), and Long-range (>200 m)
3. Type of LiDAR Devices: Stationary and Portable
4. Weight: Heavy (>6 kg), Moderate (4–6 kg), and Light (<4 kg)

**Fig. 1:**
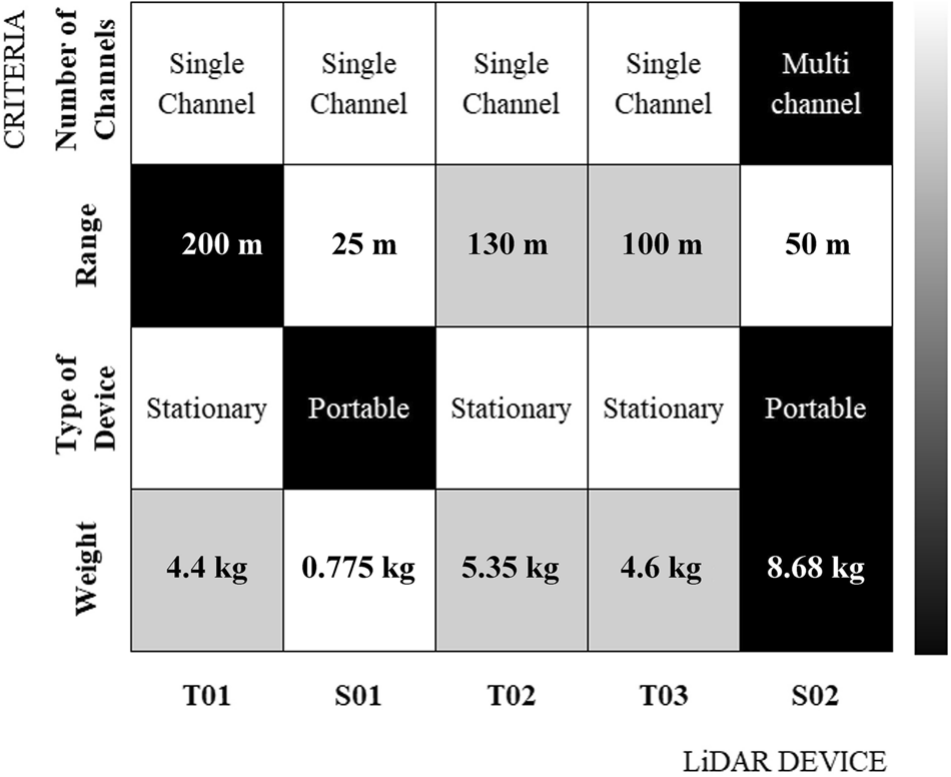
Heatmap of LiDAR Devices Characteristics. Source: Figure prepared by the authors based on LiDAR devices guides.

These categories allowed for selecting devices that represented a broad spectrum of technological capabilities, operational profiles, and practical constraints. The characteristics of each LiDAR device according to these criteria are summarized in Table 1 and visually represented in Fig. 1.

All selected LiDAR devices are classified as Class 1 according to the requirements of IEC 60825-1:2014, meaning their laser emission poses no ocular risk under normal operating conditions, ensuring safety for operators and users.

Figure 1 presents the results based on the established criteria for each selected LiDAR Device, confirming the intended diversity among them.

### Study area

The selected study area is located within Jardim Colombo, a favela in São Paulo, Brazil and spans approximately 5,685 m². The site was chosen for its morphological diversity, which includes narrow alleys, steep slopes, staircases, and zones partially or fully obstructed by buildings. These characteristics are representative of the spatial complexity commonly found in Brazilian informal settlements.

**Fig. 2:**
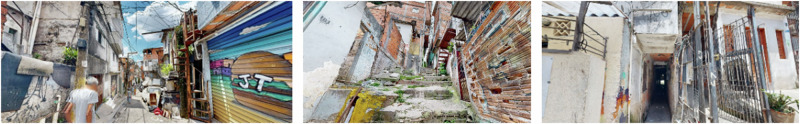
Afonso de Oliveira Santos Street, José Dias da Costa Street Stairway, and covered alley. Source: Picture by authors.

The urban layout includes mixed-use buildings, with a predominance of residential structures, as well as a religious facility and rooftops occasionally used for vehicle parking. Circulation paths vary in form and accessibility, comprising central streets, pedestrian stairways, and covered alleys. In some cases, pedestrian and vehicular traffic share the same narrow street segment (Fig. 2).

Aerial images revealed that certain pedestrian routes are obstructed or fully covered by buildings, making them invisible in aerial surveys. These conditions highlight the limitations of aerial photogrammetry in capturing the full extent of spatial configurations in favelas. Terrestrial data acquisition is therefore essential for improving map accuracy and supporting inclusive urban planning.

The case study area was selected to reflect these constraints and enable the testing of terrestrial LiDAR devices under realistic conditions typical of high-density informal urban environments.

### Field survey protocol

Field survey planning included a preliminary assessment phase to define data acquisition strategies. At this stage, both logistical and contextual factors were analyzed, including physical constraints (e.g., road conditions and equipment parking feasibility) and social considerations (e.g., security risks and community involvement in monitoring activities).

**Fig. 3:**
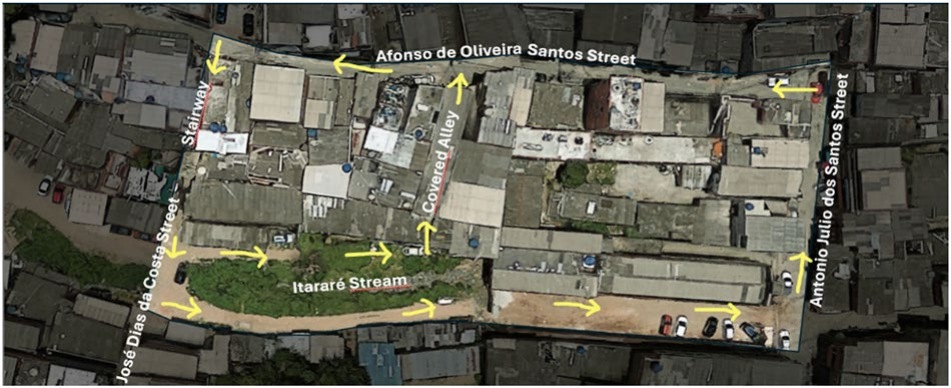
Survey route defined for LiDAR data acquisition. Source: Figure prepared by the authors based on the aerial photo.

A reconnaissance visit on October 30, 2024, was conducted to evaluate site accessibility and define the most representative and feasible path. The final route extended over 360 m and included sections with steep slopes, narrow alleys, uneven terrain, and areas with significant visual occlusions. This predefined path served as a common reference for all devices tested in the subsequent acquisition stages (Fig. 3).

### Georeferencing procedure

**Fig. 4:**
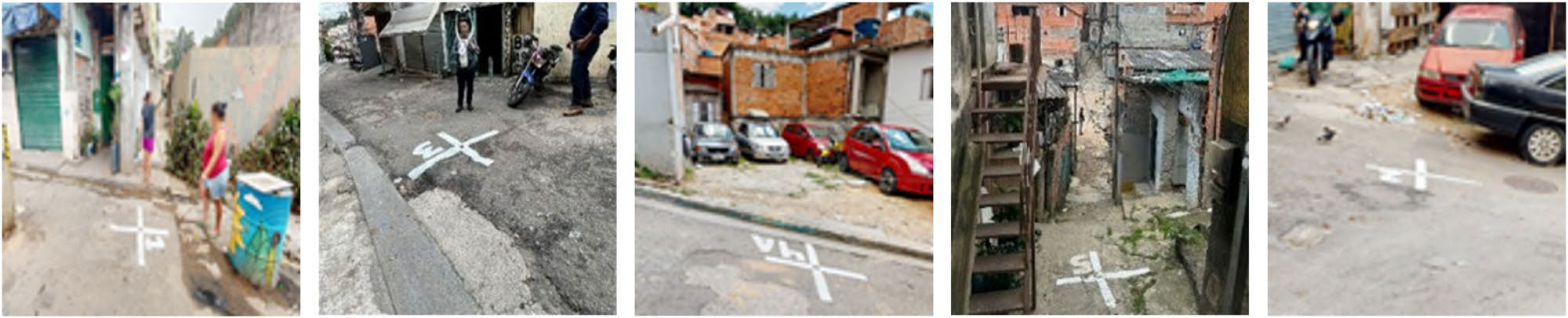
Photos of some reference points used in the georeferencing procedure. Source: Picture by authors.

**Fig. 5:**
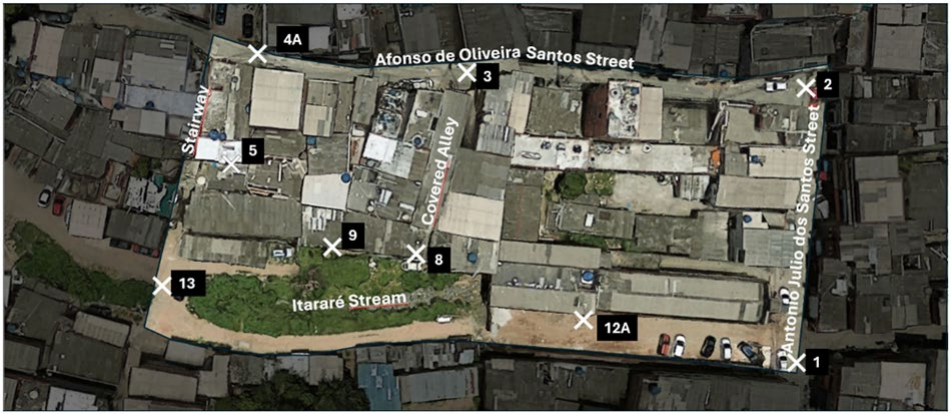
Spatial distribution of the reference points used for georeferencing. Source: Figure prepared by the authors based on the aerial photo.

To ensure accurate spatial alignment and enable reliable comparison across LiDAR datasets, a unified georeferencing procedure was applied using drone-based imagery and high-precision reference points. To support this process, three high-precision instruments were used: a total station, a GNSS receiver, and a drone. On November 5, 2024, pre-established reference points were marked in the field with white-painted crosses on the ground (Fig. 4). These points were distributed to ensure uniform coverage and visibility across the area of interest (Fig. 5). Some points were also positioned in areas with restricted visibility to ensure their integration into the topographic surface and preserve reversibility, such as stairs and alleyways obstructed by rooftops. All datasets were transformed into a common coordinate reference system (SIRGAS 2000 / UTM Zone 23S, EPSG:31983) to ensure spatial consistency and enable direct comparison across the LiDAR acquisitions.

A total of 14 reference points were surveyed in the study area. These points were used as spatial references for the georeferencing of both the drone survey and the terrestrial LiDAR datasets, ensuring that all acquisitions were aligned to the same common georeferenced base. The reference points were distributed to provide adequate coverage of the study area, including locations with restricted visibility such as stairs and narrow alleys.

To collect this data, a topographic survey was carried out with the total station and GNSS equipment on November 5 and 6, 2024. For aerial photogrammetry, two drone flights were conducted on November 12, 2024, using cross-flight paths to avoid occlusions and increase surface detail. One flight covered the North–South direction and the other the East–West direction, ensuring 80% × 80% overlap.

After data acquisition, spatial alignment consistency was checked by comparing the LiDAR point clouds with the orthophoto-derived products and the common georeferenced base. Georeferencing accuracy, however, was established primarily from the surveyed reference points measured with total station and GNSS.

### LiDAR survey

The field activities were carried out on November 5, 6, and 12, 2024, taking advantage of the opportunity to use different devices simultaneously.

On November 5, 2024, the route was surveyed using S02. On November 6, two devices covered the same route: S01 and T02. Finally, on November 12, T03 and T01 followed the same path.

To ensure an impartial comparison of the results, without factors that could compromise the analysis, all devices started from the same initial point and followed the same route. All surveys were conducted in the morning under similar lighting conditions to ensure consistency across datasets.

### Point cloud processing

The raw point clouds from the terrestrial LiDAR devices were originally provided in .e57 format, while the drone-derived dataset was supplied in. laz. All datasets were processed using CloudCompare^8^ (v.2.13.2), an open-source software for 3D point cloud visualization and analysis.

Processing steps included noise filtering, removal of outliers, and cropping to match the defined study area. No advanced post-processing procedures—such as classification, segmentation, or meshing—were applied. This minimal approach was adopted to preserve the native characteristics of each dataset and ensure that observed differences reflect variations in device performance rather than processing artifacts.

All point clouds were aligned to a unified coordinate system based on the georeferenced control points, ensuring spatial consistency across datasets. The processed datasets were subsequently saved and archived in the original formats provided: .e57 for terrestrial scans and .laz for the drone dataset. File naming conventions were standardized, and the data were organized into structured folders for public release via Zenodo.

This standardized processing pipeline ensures that the datasets are ready for scientific reuse in a variety of 3D mapping applications.

### Quality metrics: density, completeness, and uniformity

It is important to note that point cloud quality is not spatially homogeneous, even within a given sampled area. Variations in distance to the sensor, surface reflectance, incidence angle, and local geometric complexity may influence point density and positional accuracy. Therefore, no assumption of uniform quality across the dataset is made. The analysis is based on representative sample areas that capture typical acquisition conditions, providing a consistent basis for comparative evaluation.

**Fig. 6:**
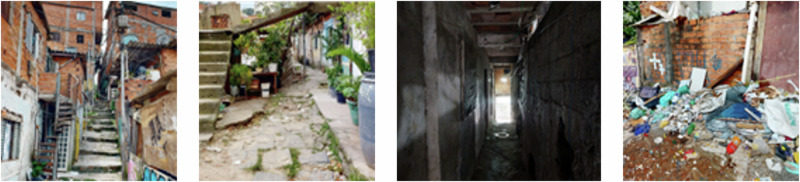
Photographs of the representative sample areas selected for evaluating point clouds. Source: Picture by authors. Note: Representative field photographs of the four sample areas (steep slopes, uneven terrain, covered alleys, and debris zones) are provided in the dataset to support interpretation of acquisition constraints and spatial occlusion conditions.

The point clouds were evaluated using three quality metrics: density, completeness, and uniformity. Density was calculated across the entire point cloud to capture overall spatial resolution. In contrast, completeness and uniformity were evaluated within four representative sample areas—steep slopes, uneven terrain, covered alleys, and zones with non-structural objects such as debris (Fig. 6).

Point clouds consist of independent points with variable spacing between adjacent points. To assess the quality of each point cloud, three main criteria were considered:

- **Density** refers to the concentration of points within a defined area; a cloud is considered denser or sparser based on the number of points per unit of area.
- **Completeness** evaluates the presence of data gaps, that is, regions with missing points, which may result from occlusions caused by obstacles blocking the LiDAR sensor’s line of sight during acquisition.
- **Uniformity** measures the regularity in point distribution, focusing on the consistency of distances between neighboring points.

In addition to these technical aspects, logistical performance was also analyzed and is presented in a separate section.

Each quality criterion was evaluated separately and scored on a three-point scale from 1 to 3, where 3 indicates high performance, 2 indicates moderate performance, and 1 indicates low performance.

To ensure comparability, the same scoring method and classification thresholds were applied across all devices.

### Density analysis

Point cloud density was calculated by dividing the total number of points by the total surveyed area, providing a global metric of spatial resolution for each dataset. Global point cloud density is therefore reported in pts/m². This value served as the basis for comparing sampling resolution across the different LiDAR point clouds.

It is important to note that the surveyed area includes buildings that could not be captured by any device. As a result, the reported densities do not represent the density of the scanned surfaces themselves, but rather a global value based on the total perimeter. This approach was adopted to allow fair comparison across all devices using a consistent area baseline.

To standardize interpretation, we applied the equal interval discretization method to the observed density values, converting them into a 1–3 point scale, as follows: 3 points (High density): values above 110,000 pts/m²; 2 points (Medium density): values between 60,000 and 110,000 pts/m²; and 1 point (Low density): values below 60,000 pts/m².

This classification enabled objective comparison of sampling density across devices, particularly in the context of heterogeneous urban environments such as favelas. Higher density was not interpreted as an absolute indicator of better point cloud quality. In this study, density was considered together with completeness, uniformity, and logistical performance as part of a broader comparative assessment.

### Completeness analysis

To evaluate the surface completeness of the point clouds, we employed the rasterization tool in *CloudCompare* to project each dataset onto a 1 cm resolution grid. A convex hull was computed from the projected cloud to define its boundary, and only the grid cells within this boundary were retained for analysis. Each occupied cell was assigned a value of 1 (blue), and each unoccupied cell a value of 0 (red). The completeness metric was then calculated as the ratio of occupied cells to the total number of cells within the boundary, expressed as a percentage.

### Completeness Index construction

To consolidate the evaluation, four sample areas were selected within the study site: steep terrain, uneven surfaces, covered alleys, and non-structural objects. Each device was independently scored on a scale from 1 to 3 based on the proportion of surface coverage derived from raster analysis. In sample areas containing more than one relevant surface, completeness was calculated separately for each surface and then averaged to obtain a mean completeness value representing the overall sampled scene. The final mean score for each LiDAR dataset was then obtained from the four sample areas and normalized using equal interval discretization across the full scoring range (1.00 to 3.00), resulting in a standardized classification: 3 points (high completeness), 2.35–3.00; 2 points (medium completeness), 1.68–2.34; and 1 point (low completeness), 1.00–1.67.

This approach allowed for a unified comparison of completeness levels across devices, reflecting their ability to capture surface detail under varied field conditions.

### Uniformity analysis

The density and spatial stability of the point clouds were evaluated using three complementary metrics: surface density, coefficient of variation, and stability index. These metrics were used as comparative indicators within the scope of this study and should be interpreted in relation to the acquisition conditions and spatial constraints of each dataset.

### Surface density

For each point cloud, local surface density was calculated using the geometric feature analysis tool in CloudCompare. This metric was not obtained by projecting a 3D volume onto a predefined horizontal or vertical surface. Instead, it was estimated locally around each point using a fixed circular neighborhood and normalized by the corresponding support area, yielding values expressed in pts/m². A fixed circular neighborhood was adopted to balance spatial sensitivity and computational efficiency.

The surface density was calculated as:

1 $${\boldsymbol{\rho }}=\frac{{\boldsymbol{N}}}{{\boldsymbol{\pi }}{{\boldsymbol{r}}}^{{\bf{2}}}}$$

where **ρ** represents the surface density, **N** is the number of points within the defined neighborhood, and **r** is the radius of the circular neighborhood.

To avoid computational bottlenecks caused by excessively large neighborhoods, a radius of approximately 0.0564 m was adopted. This radius corresponds to a circular support area of approximately 0.01 m² (100 cm²), which was used as the local neighborhood size for the density analysis.

### Mean surface density and standard deviation

Statistical parameters were computed in CloudCompare for each representative sample area. The distribution of surface density values was examined visually using histograms and fitted curves to characterize variation in point density within each sample area.

#### Coefficient of variation (CV)

The coefficient of variation (CV) was used to quantify the relative variability of surface density and was calculated as:

2 $${\boldsymbol{CV}}=\frac{{\boldsymbol{\sigma }}}{{\boldsymbol{\mu }}}$$

The coefficient of variation is a dimensionless ratio defined as the standard deviation divided by the mean, where **σ** represents the standard deviation of surface density and **μ** represents the mean surface density. When useful for interpretation, the resulting value may be expressed as a percentage. Lower CV values indicate a more homogeneous point distribution.

### Stability index (R)

To complement the analysis, a stability index **R** was used to assess the regularity of point distribution. It was calculated as:

3 $${\boldsymbol{R}}=\frac{{\boldsymbol{\mu }}}{{\boldsymbol{\sigma }}}$$

The stability index was defined as the inverse of the coefficient of variation, where **μ** represents the mean surface density and **σ** represents the standard deviation of surface density. This formulation was adopted so that lower relative variability corresponds to higher stability. Higher values of **R** therefore indicate a more homogeneous and stable spatial distribution of points.

### Scoring assignment via equal interval discretization

For each metric, values were divided into three classes using equal interval discretization, according to:

4 $${\rm{Interval}}=\frac{{\rm{MaxValue}}-{\rm{MinValue}}}{{\bf{3}}}$$

This procedure classified the results into three performance levels: 3 points (high performance), 2 points (medium performance), and 1 point (low performance).

### Uniformity score

To derive a unified uniformity indicator, the classification scores obtained for the coefficient of variation and the stability index were summed, generating a raw score for each point cloud:

5 $${\rm{RawScore}}={\rm{CVscore}}+{\rm{Rscore}}$$

This raw score was then normalized using equal interval discretization:

6 $${\rm{Interval}}=\frac{{\rm{MaxRawScore}}-{\rm{MinRawScore}}}{{\bf{3}}}$$

The resulting values were classified into three levels: 3 points (high performance), 2 points (medium performance), and 1 point (low performance).

### Uniformity index construction

To evaluate uniformity objectively, four representative areas were analyzed: steep terrain, uneven surfaces, covered alleys, and non-structural elements. For each LiDAR dataset, scores were assigned based on the regularity of point spacing and the consistency of density distribution across these areas.

The final uniformity score for each device corresponded to the average of the scores obtained in the four sample areas. These average values were then normalized into a standardized scale ranging from 1.00 to 3.00, resulting in the following classification: 3 points (high uniformity), 2.35–3.00; 2 points (medium uniformity), 1.68–2.34; and 1 point (low uniformity), 1.00–1.67.

This approach enabled a transparent and comparative assessment of spatial uniformity across all LiDAR datasets.

## Logistical Performance Index (LPI)

The Logistical Performance Index (LPI) is a study-specific metric proposed in this work to assess the operational suitability of each LiDAR device under field conditions typical of dense informal settlements. Although logistical performance is not a standardized concept in the LiDAR literature, it was introduced here to capture practical constraints that directly affect data acquisition in complex urban environments, including survey time, equipment weight, and portability.

### Survey time

Survey time was defined as the duration of data acquisition, measured from the beginning to the end of scanning, excluding setup and calibration procedures. This variable was used as an indicator of field efficiency under comparable acquisition conditions.

### Weight

Weight refers only to the portion of the equipment physically carried during field operations. Accessories not directly handled during acquisition, such as transport cases or external support items, were excluded from this analysis.

### Portability

Portability was evaluated according to the level of physical effort and operational constraint associated with each device during fieldwork. Devices were classified into three categories: 3 points (high performance), handheld devices that do not interfere significantly with the user’s walking pattern or acquisition speed; 2 points (medium performance), wearable devices that allow continuous acquisition but may restrict movement in narrow or constrained areas; and 1 point (low performance), tripod-based devices that require repeated positioning and alignment, increasing operational difficulty in steep or obstructed environments.

### Index construction

Each subcriterion, survey time, weight, and portability, was normalized using equal interval discretization based on the observed minimum and maximum values:

7 $${\rm{Interval}}=\frac{{\rm{MaxValue}}-{\rm{MinValue}}}{{\bf{3}}}$$

This procedure classified the values into three performance levels: 3 points (high performance), 2 points (medium performance), and 1 point (low performance).

For survey time and weight, the scoring was applied inversely, meaning that lower values received higher scores. The portability score was assigned directly according to the operational characteristics of each device, as described above.

The final LPI score for each device was calculated as the arithmetic mean of the three subcriteria:

8 $${LPI}=\frac{{S}_{t}+{S}_{w}+{S}_{p}}{3}$$

where **S**_**t**_ is the survey time score, **S**_**w**_ is the weight score, and **S**_**p**_ is the portability score.

Higher LPI values indicate greater operational suitability for data acquisition in complex field conditions such as those found in favelas.

This metric was not intended as a universal LiDAR quality indicator, but as a comparative operational measure tailored to the specific acquisition conditions of this study.

## Data Records

The data generated in this study are publicly available in Zenodo under the Creative Commons Attribution 4.0 International License (CC BY 4.0). Due to file size limitations, the complete dataset is distributed across two Zenodo repositories that together form a single unified dataset.

Dataset Subset A, available at 10.5281/zenodo.19324526, contains the main data products generated in this study. This repository includes the terrestrial LiDAR point clouds acquired with the five evaluated devices, the drone-derived orthophoto, the aerial point cloud used as a reference dataset, intermediate analysis files, tabular outputs, and supporting visual materials. The terrestrial point clouds are provided in .e57 format, the aerial point cloud in .laz, and the orthophoto in .tif. Intermediate analysis files are provided in CloudCompare .bin format, tabular outputs are provided in .csv and .xlsx formats, and supporting visual materials, including representative images and validation graphics, are provided in .png or .jpg formats.

Dataset Subset B, available at 10.5281/zenodo.15164836, contains additional CloudCompare workspace files in .bin format corresponding to the Leica RTC360 dataset, including the four sample-area subsets used in the technical analysis. These files were deposited separately because of repository size limitations. Together, Subset A and Subset B provide the complete data package required to support reuse, inspection, and independent verification of the study.

The repositories are organized according to the main data categories and processing stages. At the top level, the folder structure distinguishes aerial photogrammetry products, terrestrial LiDAR point clouds for each device, intermediate analysis files, tabular outputs, and supporting visual documentation. Folder and file names were standardized to identify the device, data type, and analysis stage.

The main terrestrial LiDAR datasets are summarized in Table 2, and the aerial photogrammetry dataset is summarized in Table 3. In addition to the raw point cloud products, the repositories include analysis-ready files used during the evaluation workflow, such as CloudCompare workspace files, derived raster and image outputs, and tabular records associated with density, completeness, uniformity, and logistical analyses. Although these files are not primary data products, they are provided to support procedural traceability, transparency, and independent verification of the reported results.

**Table 2:** Data records for the terrestrial LiDAR datasets.

Device	T01	S01	T02	T03	S02
**Representative visualization**					
**File name**	PC_Faro	PC_LeicaBLK	PC_LeicaRTC	PC_Matterport	C_NavVis
**Size** (gigabytes)	16.9	2.01	18.6	11.0	4.81
**Folder in Zenodo**	00b_PC_T1 _Faro	00b_PC_S1 _LeicaBLK	00b_PC_T2 _LeicaRTC	00b_PC_T3 _Matterport	00b_PC_S2 _NavVis
**File type**	.e57	.e57	.e57	.e57	.e57

Source: Authors.

Note: Representative visualizations illustrate the terrestrial point cloud datasets acquired with each LiDAR device.

**Table 3:** Data records for the aerial photogrammetry dataset.

Device	Drone	
**Content**	Orthophoto	Point Cloud
**Image visualization**		
**File name**	Drone_Orthophoto	Drone_Point Cloud
**Folder in Zenodo**	00a_PC_DroneM3E	00a_PC_DroneM3E
**Size** (gigabytes)	80.5	128
**File type**	.tif	.laz

Source: Authors.

Note: The orthophoto and 3D point cloud generated by drone are shown.

The tabular outputs document both intermediate and final results of the technical evaluation. These files include, for example, device-specific metrics, scores assigned to each representative sample area, and consolidated values used to derive the classifications reported in the manuscript. Where column names or worksheet fields are not immediately self-explanatory, the corresponding file names, worksheet organization, and repository documentation indicate the associated device, metric, or sample area.

The repositories also include RGB photographs documenting the study area, representative sample areas, reference points used in georeferencing, and field survey activities. These images provide visual context for the surveyed environment and support interpretation and reuse of the LiDAR datasets, particularly in relation to acquisition constraints, occlusions, and spatial conditions observed in the study area.

A README file included in the Zenodo repositories provides additional documentation on folder organization, file contents, naming conventions, and the meaning of non-implicit tabular fields. This file should be consulted together with the present Data Records section to identify the relevant products for reuse.

All datasets are provided in open or widely used formats and organized to facilitate reuse in studies involving urban morphology, infrastructure analysis, cartographic interpretation, and methodological comparison of LiDAR acquisition in complex informal settlements.

## Technical Validation

### Georeferencing

Georeferencing accuracy was assessed by comparing each LiDAR dataset with the common georeferenced base generated from the surveyed reference points measured with total station and GNSS. Orthophoto-derived products were used only as complementary support for checking spatial alignment consistency. Residual errors were calculated using only the reference points that were effectively visible and usable in each point cloud. Because visibility varied across datasets due to occlusions and acquisition geometry, not all surveyed reference points could be used in the validation of every LiDAR dataset. For this reason, Table 4 reports only the reference points effectively used in the validation of each dataset, together with the final RMS value for each device.

**Table 4:** Residual errors at surveyed reference points used for georeferencing validation.

Reference point	T01	S01	T02	T03	S02
1	0.0218105	0.129833	0.0146048	0.0732793	0.0217163
1ª	0.0157146	0.365075	0.0243029	0.0282508	0.0750309
2	0.0111486	0.303505	0.00635059	0.0538685	0.0405373
3	0.00264501	0.0751202	0.0148635	0.1101	0.109846
4ª	0.00705293	0.232552	0.00524831	0.0685937	0.0173651
5	0.0313826	0.0712221	0.00407978	0.0434888	0.0344247
9	0.0055084	0.207906	0.008463	0.0357966	0.0379414
8	—	0.227671	0.00519409	0.0490574	0.0442563
Final RMS (m)	0.0165568	0.224195	0.0122881	0.0627259	0.05556

Source: Authors

**Note:** A total of 14 reference points were surveyed in the study area and used as spatial references for the georeferencing of both the drone survey and the terrestrial LiDAR datasets, ensuring a common georeferenced base for the study. Table 4 reports residual errors only for the reference points effectively visible and usable in the validation of each LiDAR dataset. Not all surveyed points were available in all datasets because of occlusions and acquisition geometry. Residual values are expressed in meters.

### Density, completeness, and uniformity analysis

Table 5 summarizes the results of the analyses of the point clouds obtained from the terrestrial LiDAR devices.

**Table 5:** Density, completeness, uniformity, and logistical analysis of the terrestrial LiDAR datasets.

Density Analysis	T01	S01	T02	T03	S02
Total Points	646,867,758	111,174,505	1,043,868,712	340,966,867	127,632,980
Area (m²)	5,685	5,685	5,685	5,685	5,685
Density (pts/m²)	113,785	19,556	183,618	59,976	22,452
Final Density Classification	3	1	3	1	1
**Completeness Analysis**	**T01**	**S01**	**T02**	**T03**	**S02**
Sample Area: Steep Slopes	3	1	2	3	2
Sample Area: Uneven Terrain	2	1	2	3	1
Sample Area: Covered Alleys	3	2	1	3	2
Sample Area: Debris	3	1	3	3	3
Mean Score	2.75	1.25	2.00	3.00	2.00
Final Completeness Classification	3	1	2	3	2
**Uniformity Analysis**	**T01**	**S01**	**T02**	**T03**	**S02**
Sample Area: Steep Slopes	3	1	1	1	3
Sample Area: Uneven Terrain	2	1	1	1	3
Sample Area: Covered Alleys	2	1	1	1	3
Sample Area: Debris	3	1	3	2	3
Mean Score	2.50	1.00	1.50	1.25	3.00
Final Uniformity Classification	3	1	1	1	3
**Logistical Analysis**	**T01**	**S01**	**T02**	**T03**	**S02**
Time Spent in the Field	5 h 10 min	30 min	3 h	2 h 45 min	45 min
Time Classification	1	3	2	2	3
Weight (kg)	4.2	0.775	5.1	2.3	8.7
Weight Classification	2	3	1	2	1
Portability	1	3	1	1	2
LPI (Mean Score)	1.33	3.00	1.33	1.67	2.00
Final Logistical Classification	1	3	1	2	2

Source: Authors.

Note: Density values are expressed in points per square meter (pts/m²). Completeness and uniformity scores were derived from four representative sample areas and normalized using equal interval discretization into a three-point scale (1–3), where 3 indicates high performance and 1 indicates low performance.

### Synthesis of the analysis

An overview of all evaluation criteria is summarized in Table 6. Device T01 obtained the highest scores in point cloud quality (density, completeness, and uniformity), while S01 performed best in terms of logistical practicality.

**Table 6:** Synthesis of the analysis.

Final Classification	T01	S01	T02	T03	S02
Density	3	1	3	1	1
Completeness Analysis	3	1	2	3	2
Uniformity Analysis	3	1	1	1	3
Logistical Analysis	1	3	1	2	2
Final Score	2.50	1.50	1.75	1.75	2.00
Final Classification	High Performance	Low Performance	Moderate Performance	Moderate Performance	Moderate Performance

Source: Authors.

Note: The Logistical Performance Index (LPI) represents the arithmetic mean of three normalized subcriteria: survey time, equipment weight, and portability. Each subcriterion was classified on a three-point scale (1–3) using equal interval discretization, except for portability, which was assigned based on operational characteristics.

All LiDAR datasets were collected along the same predefined survey route within the study area, ensuring that each device captured the same urban environment under comparable spatial conditions. This strategy minimizes variations related to acquisition geometry and enables consistent comparison of density, completeness, and uniformity metrics across devices.

To further ensure comparability, all datasets were georeferenced to the same coordinate reference system and analyzed using identical processing parameters within the CloudCompare environment.

To support transparency, procedural traceability, and independent verification, intermediate analysis files in CloudCompare (.bin) format are also provided in the Zenodo repository, including spatial subsets and intermediate metric calculations used during the evaluation process. These files allow direct inspection of the procedures used to compute the metrics and classification scores reported in the tables. This approach supports independent verification and transparent comparison of LiDAR datasets across devices.

Technical validation was performed through the computation of density, completeness, and uniformity metrics derived from the terrestrial LiDAR point clouds. To support transparency, procedural traceability, and independent verification, the Zenodo repository includes not only the raw point clouds but also the intermediate analysis files used during the evaluation process, including CloudCompare workspace files (.bin), intermediate raster and image outputs, and tabular datasets containing the computed metrics. These materials allow researchers to inspect the analytical workflow, verify the reported results, and reuse the processed datasets in further analyses. All intermediate files used in the analysis pipeline are publicly available together with the raw datasets. The point clouds are provided in standard open formats (.e57 and .laz), ensuring compatibility with commonly used point cloud processing software. Because some processing steps were performed manually, exact replication of the full workflow may depend on user decisions made during interactive editing.

## Usage Notes

The point cloud datasets provided in this study are high-density and represent a complex urban environment. As such, processing these files may require computers with sufficient computational power and memory, particularly when working with full-resolution data.

We recommend using CloudCompare (v.2.13.2), an open-source 3D point cloud processing software, for data cleaning, visualization, and geometric analysis. For spatial integration, QGIS or similar GIS platforms can be used to overlay the point clouds with georeferenced base layers.

The datasets are available in standard formats—.e57 for raw terrestrial LiDAR, .laz for drone-based data—and in CloudCompare’s native.bin format, which contains all processed versions used in the analysis. These.bin files preserve the structure and annotations created during processing and are useful for users wishing to inspect, verify, reuse, or extend the study workflow.

All datasets are compatible with most LiDAR and GIS software. When integrating them with external geospatial sources (e.g., official cartographic portals), users should ensure coordinate system compatibility, as all data are georeferenced in SIRGAS 2000 / UTM Zone 23S.

No additional processing has been applied to the raw point clouds beyond basic cleaning. Users may apply registration, filtering, classification, or meshing as needed, depending on their specific research objectives.

## Data Availability

The datasets generated in this study are publicly available in Zenodo under the Creative Commons Attribution 4.0 International License. Due to file size limitations, the complete dataset is distributed across two repositories. Dataset Subset A, containing the main terrestrial LiDAR datasets, the aerial photogrammetry products, and the principal intermediate analysis files, is available at 10.5281/zenodo.19324526^9^. Dataset Subset B, containing additional CloudCompare workspace files related to the Leica RTC360 dataset, is available at 10.5281/zenodo.15164836^10^. Detailed descriptions of file contents, formats, and folder organization are provided in the Data Records section.

## Code availability

No custom code was used to generate or process the dataset described in this manuscript. All point cloud processing was performed using CloudCompare (v.2.13.2), an open-source software for 3D point cloud editing and analysis.

All cleaning, cropping, and visualization steps were executed manually through the software’s graphical user interface, without the use of automation scripts or plugins. The processed datasets are provided in CloudCompare’s native.bin format, which preserves the full project structure, including coordinate transformations, clipping boundaries, and other processing history.

This format supports transparency, procedural traceability, and independent verification of the reported analyses. However, because some processing steps were performed manually through the graphical user interface, exact replication of the full workflow may depend on user decisions made during interactive editing.
